# Self-Reported Time to Diagnosis and Proportions of Rediagnosis in Female Patients with Chronic Conditions in Australia: A Cross-sectional Survey

**DOI:** 10.1089/whr.2022.0040

**Published:** 2022-09-12

**Authors:** Lea Merone, Komla Tsey, Darren Russell, Andrew Daltry, Cate Nagle

**Affiliations:** ^1^ College of Healthcare Sciences, James Cook University, Townsville, Australia.; ^2^ College of Arts, Society and Education, James Cook University, Smithfield, Australia.; ^3^ Cairns Sexual Health Service, Cairns North, Australia.; ^4^ Cairns and Hinterland Hospital and Health Service, Cairns, Australia.

**Keywords:** diagnosis, chronic conditions, women's health, misdiagnosis

## Abstract

**Background:**

The diagnosis of chronic conditions in women is complicated by the historical androcentricity in medical research. Sex and gender gaps in health research may translate to unequal healthcare for women. This cross-sectional survey study aimed to ascertain the median time to diagnosis, proportions of rediagnosis and time to rediagnosis for Australian women with chronic conditions.

**Methods:**

An online survey collected anonymous data from voluntary participants. Data were analyzed using Stata14. Cox Proportional Hazards model was used to analyze time to diagnosis and rediagnosis. Logistic regression analysis was used to assess the significance of rediagnosis rates by diagnosis, age at diagnosis, income, employment, state of residence, disability status, and Indigenous status.

**Results:**

The median time from first appointment to initial diagnosis was 6 months (range 1 day–50 years) (interquartile range [IQR] 3.74 years). The median time to rediagnosis was 4 years (IQR 9) with a range of 1 day–43 years. Almost half of the women (*n* = 161/343, 47%) reported their primary condition being rediagnosed. From the complete responses, 40% were rediagnosed from one organic condition to another organic condition, however, 32% of women originally diagnosed with psychological, medically unexplained syndromes, or chronic pain were later rediagnosed with organic conditions.

**Conclusion:**

Median wait times for a diagnosis for women in Australia, when factoring in high rates of rediagnosis and time to rediagnosis, was 4 years. It is important that clinicians are aware of the high rediagnosis rates in female patients with chronic conditions and understand the potential impact of systemic biases on the diagnostic process for women under their care.

## Background

Diagnosis of disease in women is complicated by the androcentric history of medical research,^1^ and systemic and societal gender biases.^2,3^ Previously, women's health has been under-researched, and results obtained from studies of male bodies have been assumed to apply to females.^4,5^ Assuming females and males to be the same is flawed and studies have demonstrated physiological,^6^ hormonal,^7,8^ microbiotal,^9^ and socioeconomic differences^10^ that impact upon women's health, symptoms,^11^ test results, and responses to treatment.^12^ Despite this, many studies continue not recruiting enough women^13^ and many more studies do not analyze their results by sex and/or gender.^14^

A recent cross-sectional analysis demonstrated that while across all clinical research in Australia, female participation was 55%, when analyzed by medical specialty, certain specialty areas over- and under-represented women.^13^ Perception of disease, rather than actual sex prevalence may drive representation of women in medical research.^15^ If this is the case, it could become a self-fulfilling prophecy, whereby under-diagnosis of some conditions in women enhances the perception of those conditions as male dominated, leading to lower recruitment of women into clinical trials pertaining to that condition.

Conditions with female predominance such as fibromyalgia^16^; myalgic encephalomyelitis/chronic fatigue syndrome (ME/CFS)^17^; autoimmune conditions, for example, systemic and cutaneous lupus erythematosus (collectively referred to from herein as lupus) and rheumatoid arthritis^18^; postural orthostatic tachycardia syndrome (POTS)^19^; vulvodynia; and endometriosis receive less research funding than diseases, such as heart disease, cancers, dementia, and—more recently—coronaviruses.^20^ Lack of funding has ongoing impacts on clinical knowledge regarding pathophysiology, presentation, treatment options, and responses to treatments that may have significant impact on women's health.^21^

Additionally, several of these female-dominated conditions are considered to be diagnoses of exclusion when all radiology and laboratory results are normal or nonspecific.^22^ These conditions may be termed as “psychogenic,” “medically unexplained symptoms” (MUS), or “functional”.^23^ The reality of these conditions may well be that owing to limited research funding, we simply do not yet know the pathogenesis, rather than the conditions being largely or entirely psychological in origin. Gender biases may also enhance the appearance of some conditions as being female dominated. Katz et al surveyed American rheumatologists and uncovered a gender bias in the diagnosis of fibromyalgia, with physicians being more likely to seek a more accepted organic disease in male patients.^3^

Studies have shown that sex and gender differences are also largely excluded from medical education materials, such as clinical and anatomical textbooks and medical school course outlines and curricula.^12,24–27^ A previous review of sex/gender representation in anatomy textbooks determined that imagery remains male centered.^26^ Consequently, students may come to view the male body as the norm, and the female body as “abnormal.” Additionally, students may become less confident with female anatomy and examinations.^28,29^ Descriptions of women's symptoms as “atypical” compared with those of men exemplifies androcentricity in medicine and research.^12^

The sex and gender gaps in research and education may translate into real-life impacts and unequal health care for women. There are notable sex and gender differences in acute care; women wait longer in the Emergency Department for a diagnosis^30^ and for adequate analgesia.^31^ A large study outside of Australia noted that women wait longer than men for a diagnosis.^32^ Internationally, women are noted to frequently be misdiagnosed and then later rediagnosed.^33^ The average time to diagnosis and rates of rediagnoses are not yet documented for Australia and most studies pertain to individual, specific conditions rather than general chronic conditions. This study aimed to ascertain the median time from presentation to initial diagnosis, the proportion of women who were rediagnosed, the median time to rediagnosis and the impact of socioeconomic factors on time to diagnosis and rediagnosis.

## Methods

A cross-sectional survey of Australian women was conducted online, hosted on the survey software *Qualtrics*. The survey was devised using guidelines from Kelly and Gurr; Valerie and Ritter and Letherby.^31–33^ All data collected were anonymous. Adults 18 years of age and older who were born and reside in Australia were included. Participants unable to understand and respond to written English were not included. The following data were collected: initial diagnosis; age at initial diagnosis; rediagnosis; time to rediagnosis; income; employment; state/territory of residence; Indigenous status; disability status; current age; and secondary diagnoses. A summary of the data collection tool is provided in Box 1.

Initial or primary diagnosis was defined as the first diagnosis that an individual received, and rediagnosis was defined as the ultimate diagnosis a condition was given, noting that some individuals had their condition rediagnosed multiple times before an ultimate diagnosis (at the time of survey) was received. Secondary diagnoses were defined as any separate diagnoses received after and alongside the initial diagnosis.

### Data collection

Participants were recruited by convenience sampling *via* social media (Twitter and Facebook). The study was advertised using relevant hashtags and notices in relevant Australian support groups of chronic conditions, including: chronic pain; endometriosis; lupus; mast cell activation syndrome (MCAS); epilepsy; asthma; cancer; ME/CFS; fibromyalgia; depression; anxiety; Ehlers–Danlos syndrome (EDS); POTS; disability; and many more. Before completing the survey, participants were informed *via* the information leaflet that the topics discussed were sensitive and may cause some distress and subsequently that they may withdraw at any point until their survey is submitted. Human Research Ethics Committee approval from the James Cook University was obtained (H8547), and all participants provided informed consent.

### Data analysis

The primary outcomes were the self-reported diagnosis, time to primary diagnosis and rediagnosis, and proportions thereof, in participants with one or more chronic conditions. The secondary outcomes were the associations of the primary outcomes with diagnosis, age at diagnosis, income, employment, disability status, and Indigenous status.

Data were analyzed using Microsoft Excel and the statistical software package Stata14. Descriptive statistics were used to summarize the data. To analyze time to event (diagnosis and rediagnosis) and association of any variables (diagnosis, age, income, employment, state/territory of residence, Indigenous status, and disability status), the Cox Proportional Hazards Model^34^ was used. Logistic regression analysis was used to assess significance of rediagnosis rates by diagnosis, age at diagnosis, income, employment, state of residence, disability status, and Indigenous status. Diagnoses were analyzed specifically if more than five people received the same label, and if fewer than five people were diagnosed with a condition, these were grouped by medical specialty.

Conditions that were very rare or unable to be categorized (*e.g.*, a symptom rather than a diagnosis given) were grouped together as “unclear” or “rare.” As the diagnosis with the most definitive tests of all the common chronic conditions diagnosed in this sample, type 2 diabetes mellitus (T2DM) was selected as the reference group. Diagnoses were then grouped by the researchers into two categories, medical conditions that are traditionally considered to be “organic” such as heart disease, diabetes, autoimmune disease, and medical conditions that are traditionally considered to be psychological/psychosomatic, medically unexplained syndromes (MUS), or idiopathic chronic pain, such as ME/CFS, fibromyalgia, chronic regional pain syndrome, and mental illnesses. Researchers then assessed the proportion of “organic” diagnoses that were rediagnosed to MUS diagnoses and vice versa.

Regarding the question of rediagnosis, the survey contained three response options: “yes, the condition was rediagnosed,” “no, the condition was not rediagnosed” and “unsure.” Those unsure about their rediagnosis status were categorized as “no” for the purpose of this analysis with the rationale that there was no definitive rediagnosis timeline, and if an individual's condition had been fully rediagnosed, it was assumed the individual would be aware.

## Results

There was a total of 467 responses. The demographic details of participants are summarized in Table 1.

**Table 1.** Participants' Demographic Characteristics

Demographic characteristics	Number of participants (%)
Indigenous status	
Aboriginal	15 (3)
Torres Strait Islander	2 (0.4)
Aboriginal and Torres Strait Islander	2 (0.4)
Non-Indigenous	435 (93)
Not stated	13 (2)
State/territory of residence	
Australian Capital Territory	20 (4)
New South Wales	120 (26)
South Australia	37 (8)
Queensland	96 (21)
Northern Territory	9 (2)
Tasmania	19 (4)
West Australia	25 (5)
Victoria	139 (30)
Not stated	2 (0.4)
Employment status	
Disability support or pension	82 (18)
Full-time employed	196 (42)
Part-time employed	2 (0.4)
Self employed	41 (9)
Unemployed	31 (7)
Temporary employment	10 (2)
Retired	27 (6)
Full-time carer	7 (1)
Student	16 (3)
Other	50 (11)
Not stated	5 (1)
Current age range (years)	
18–25	13 (3)
26–35	104 (22)
36–45	121 (26)
46–55	129 (28)
56–65	72 (15)
66–75	18 (4)
76+	3 (0.6)
Not stated	7 (1)
Household weekly income after tax (AUD)	
0–743	145 (31)
744–1431	121 (26)
1432–2433	104 (22)
2434+	87 (19)
Not stated	10 (2)
Disability status	
Disabled	182 (39)
Not disabled	186 (40)
Unsure	97 (21)
Not stated	2 (0.4)
Age range at initial diagnosis (years)	
18–25	142 (30)
26–35	97 (21)
36–45	62 (13)
46–55	36 (8)
56–65	8 (2)
66–75	1 (0.2)
76+	0 (0)
Not stated	121 (26)

**Box 1.** Summarized Data Collection Tool

Age (years) Indigenous status State/Territory of current residence Name of closest town/city Employment status Household Income bracket (AUD) per week (after tax) Disability status/Identity First diagnosis (the one that was diagnosed chronologically first) Specialist who cares for first diagnosis (*e.g*., cardiologist). Number of other diagnoses List of other diagnoses Age at first diagnosis Time from first presentation with symptoms to first diagnosis Has the first condition been rediagnosed? Number of times the diagnosis changed Time from first diagnosis to rediagnosis Name of rediagnosed condition

### Diagnoses

Initial diagnoses were extremely varied and included common conditions such as asthma, T2DM, cardiac conditions, arthritis, and mental illnesses and some very rare conditions such as VACTERL syndrome, narcolepsy, Scheuermann's disease, and hidradenitis suppurativa. Eleven diagnoses were unclear or listed a symptoms, rather than a diagnosis. The most common 12 conditions reported as an initial diagnosis are presented in Table 2. These 12 diagnoses made up 168 (52%) of 325 responses to this question. The remaining conditions were grouped by specialty using classifications in the *International classification of diseases* (ICD-10),^35^ for analysis (Table 3).

**Table 2.** Most Common Initial Diagnoses

Initial diagnosis	Number of participants reporting (% of total) ***n*** = 325
Endometriosis/adenomyosis	29 (9)
Fibromyalgia	27 (8)
ME/CFS	24 (7)
SLE/cutaneous lupus/MCTD	18 (5)
Autoimmune arthritis, including RA and psoriatic arthritis	12 (4)
Depression	11 (4)
POTS	10 (3)
Chronic pain	9 (3)
Anxiety	9 (3)
IBS	9 (3)
Chronic tonsillitis	5 (2)
T2DM	5 (2)

IBS, irritable bowel syndrome; MCTD, mixed connective tissue disease; ME/CFS, myalgic encephalomyelitis/chronic fatigue syndrome; POTS, postural orthostatic tachycardia syndrome; RA, rheumatoid arthritis; SLE, systemic lupus; T2DM, type 2 diabetes mellitus.

**Table 3.** Grouped Conditions

Diagnosis (grouped by specialty)	Number of participants reporting (% of total, ***n*** = 325)
MSK conditions	29 (9)
Neurological conditions	18 (5)
Cardiac conditions	14 (4)
Unclear	12 (3)
Psychiatric conditions	11 (3)
Gastrological conditions	10 (3)
Thyroid conditions	9 (3)
Gynecological conditions	9 (3)
Hematological conditions	8 (2)
Respiratory conditions	8 (2)
Dermatological conditions	8 (2)
Infective/viral conditions	5 (2)
Endocrine conditions	4 (1)
Cancers	4 (1)
Renal conditions	4 (1)
Rare conditions	4 (1)

MSK, musculoskeletal.

The median number of secondary diagnoses (in addition to the initial diagnosis) was 3 (interquartile range [IQR] 3, range 0–20). Secondary diagnoses were wide ranging and included EDS, lupus, mental illness, MCAS, fibromyalgia, ME/CFS, endometriosis, osteopenia, irritable bowel syndrome (IBS), inflammatory bowel disease, and cancers.

### Time to initial diagnosis

**FIG. 1.**
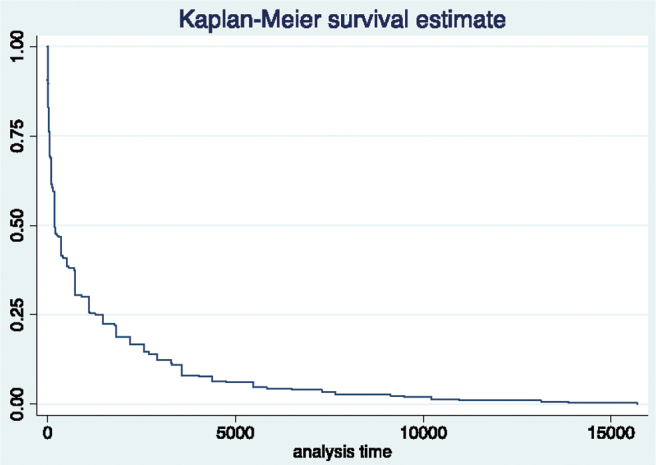
Kaplan–Meier survival estimate for event: time from presentation to a doctor with symptoms to initial diagnosis of the condition. Demonstrating time to diagnosis in days and proportion of women who received their diagnosis within this time.

The median and modal for time from first appointment to initial diagnosis was 6 months (range 1 day–50 years) (IQR 3.74 years). Kaplan–Meier survival analysis demonstrated 25% of participants received an initial diagnosis within 56 days (2 months), 50% by 168 days (6 months), and 75% by 1460 days (4 years) (Fig. 1).

Initial diagnoses that significantly delayed time to diagnosis were: anxiety, IBS, autoimmune arthritis, ME/CFS, migraine, endometriosis, fibromyalgia, chronic pain syndromes, lupus/mixed connective tissue disease, POTS, mental illness (category), cancers, neurological conditions, gastrological conditions, musculoskeletal conditions, thyroid conditions, gynecological conditions and cardiovascular conditions (Table 4). No initial diagnoses significantly shortened time to diagnosis. The only demographics to significantly impact the time to initial diagnosis were age bracket 36–45 years, which lengthened this time (coefficient −0.4228682, *p* = 0.05) and being on disability support pension or equivalent, which shortened the time (coefficient 0.4835686, *p* = 0.05). These results are summarized in the Supplementary Data.

**Table 4.** Diagnoses That Significantly Delayed Time to Obtaining That Diagnosis in Surveyed Women with Chronic Conditions in Australia

Diagnosis	Coefficient	SE	***z***	***p*** > ***z***	95% CI
Anxiety	−1.340	0.614	−2.18	**0.029**	−2.544 to −0.137
IBS	−2.711	0.625	−4.33	**0.000**	−3.937 to −1.485
Autoimmune arthritis	−1.949	0.559	−3.49	**0.000**	−3.045 to −0.854
ME/CFS	−2.797	0.526	−5.32	**0.000**	−3.828 to −1.766
Migraine	−2.039	0.589	−3.46	**0.001**	−3.192 to −0.885
Endometriosis	−3.126	0.536	−5.83	**0.000**	−4.177 to −2.076
Fibromyalgia	−2.720	0.520	−5.23	**0.000**	−3.739 to −1.701
Chronic pain	−2.444	0.629	−3.88	**0.000**	−3.677 to −1.211
Lupus/MCTD	−2.273	0.535	−4.25	**0.000**	−3.321 to −1.225
POTS	−2.619	0.640	−4.09	**0.000**	−3.874 to −1.363
Mental illness	−2.402	0.726	−3.31	**0.001**	−3.825 to −0.978
Cancers	−3.192	0.287	−2.48	**0.013**	−5.714 to −0.669
Neurological	−2.370	0.564	−4.20	**0.000**	−3.476 to −1.265
Gastrological	−2.354	0.613	−3.84	**0.000**	−3.555 to −1.153
MSK	−2.311	0.553	−4.18	**0.000**	−3.394 to −1.228
Thyroid	−2.036	0.630	−3.23	**0.001**	−3.271 to −0.801
Gynecological	−2.449	0.587	−4.17	**0.000**	−3.600 to −1.297
Miscellaneous	−2.021	0.567	−3.57	**0.000**	−3.132 to −0.910
Infective/viral	−1.664	0.663	−2.51	**0.012**	−2.964 to −0.364
Cardiology	−2.041	0.668	−3.06	**0.002**	−3.351 to −0.732

CI, confidence interval; SE, standard error.

### Rediagnosis rate

Almost half of the women (*n* = 161/343, 47%) reported their primary condition being rediagnosed, a further 36 women were unsure if their condition had been rediagnosed or not (10%). Of those 161 women who had their primary condition rediagnosed, 58 (36%) women stated this had happened three or more times. Rediagnosis rates are presented in Table 5.

**Table 5.** Rediagnosis Rates in Surveyed Women with Chronic Conditions in Australia

Variable	Proportion	SE	95% CI
Rediagnosed	0.47	0.269	0.417–0.5226
Not rediagnosed	0.42	0.267	0.374–0.479
Unsure	0.11	0.166	0.076–0.142

Diagnosis, age at diagnosis, income, employment, state/territory of residence, disability status, and Indigenous status had no effect on rates of rediagnosis, however, residing in Tasmania was approaching significance (coefficient −1.67147, *p* = 0.06) (the Supplementary Data).

### Time to rediagnosis

**FIG. 2.**
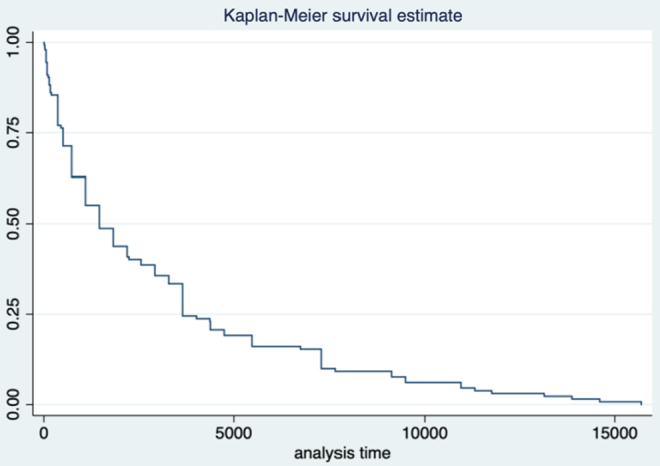
Kaplan–Meier survival estimate for event: time to rediagnosis. Demonstrating time to rediagnosis in days and proportion of women who received their rediagnosis within this time.

The median time to rediagnosis was 4 years (IQR 9) with a modal value of 1 year (range 1 day–43 years). Survival analysis demonstrated 25% of participants received a rediagnosis within 504 days (18 months), 50% within 1460 days (4 years), and 75% by 3650 days (10 years) (Fig. 2).

Of the 128 respondents who gave complete responses, including their original diagnosis and their rediagnosis, 40% (*n* = 51) were rediagnosed from one organic condition to another organic condition, however, 32% of women originally diagnosed with psychological, MUS, or chronic pain were later rediagnosed with organic conditions (Table 6). Diagnosis, age at diagnosis, income, employment, state/territory of residence, and disability status had no significant effect on time to rediagnosis, however, identifying as Torres Strait Islander was significantly associated with shorter time to rediagnosis (Supplementary Data), however, this is based on a sample of two participants.

**Table 6.** Rediagnoses of Surveyed Women with Chronic Conditions in Australia Demonstrating the Number and Percentage of Women Who Were Initially Diagnosed with One Either Organic or Psychological/Medically Unexplained Syndromes Condition and Then Later Rediagnosed with a Different Condition

Primary diagnosis group	Rediagnosis group	Number (%) ***n*** = 128
Psychological, MUS, or chronic pain condition	Organic condition	41 (32)
Organic condition	Psychological, MUS, or chronic pain condition	17 (13)
Psychological, MUS, or chronic pain condition	Another psychological, MUS, or chronic pain condition	19 (15)
Organic condition	Another organic condition	51 (40)

MUS, medically unexplained syndromes.

## Discussion

Participants typically obtained an initial diagnosis within 6 months, however, 47% of women reported rediagnosis and a median period of 4 years between initial diagnosis and rediagnosis, indicating women are potentially waiting a long time for adequate treatment and risk progression of their condition. Additionally, there are potential risks associated with receiving treatment for an incorrect initial diagnosis.

### Australian women with chronic disease participating in an online survey receive an initial diagnosis within 6 months

Our study determined a median diagnostic time of 6 months for women with chronic conditions in Australia, however, this ranged between 1 day and 50 years, and was accompanied by a high rate of rediagnoses, suggesting misdiagnosis of the initial condition. It is unclear why the 35–46-year age group at diagnosis was significantly associated with shorter time to diagnosis.

It is difficult to assess if 6 months is a lengthy duration to diagnosis as there are no comparative data on male times to diagnosis. A U.K. study defined a delay in cervical cancer diagnosis as a duration longer than 3 months from first presentation with symptoms to diagnosis.^36^ Lim et al observed a delayed diagnosis in 60% of symptomatic women.^36^ Another study noted delays in diagnoses for female patients with cystic fibrosis; compared with males, females experienced a 4-month delay in diagnosis in a large study of 11,275 cystic fibrosis patients.^37^ Conversely, cross-sectional analysis of 7,101 individuals with diabetes in Canada determined that proportionally more males are diagnosed late than females.^38^

### Almost half of Australian women with chronic disease participating in an online survey have their initial condition rediagnosed at least once

This study ascertained that almost half of female patients with chronic disease are rediagnosed for at least one occasion. This is in keeping with findings from other studies. Focus groups with fibromyalgia patients report a long and stressful journey to diagnosis with high misdiagnosis rates.^39^ The 2012 National Health Interview Survey estimated that fibromyalgia is misdiagnosed, using surrogate markers, in about three quarters of respondents.^40^ According to the Lupus Foundation of America, a lupus (encompassing four different types of lupus: systemic, cutaneous, drug-induced and neonatal) diagnosis takes on average almost 6 years from first developing symptoms, with a high misdiagnosis or rediagnosis rate and an average of four different health care providers.^41^ This was supported by a 2018 U.K. survey of lupus patients, demonstrating an average time to diagnosis of 6.4 years, with 47% initially being misdiagnosed.

Our findings of high rediagnosis rates are in keeping with Geraghty and Blease, who found that 40% of referrals to one ME/CFS clinic were eventually diagnosed with another chronic or psychiatric illness.^42^ This narrative review of the literature also determined that patients with ME/CFS report frequent misdiagnosis of their condition, possibly related to guidelines that recommend against overinvestigation of ME/CFS patients as a drain on resources, leading to underdiagnosis.^42^ A review of 418 referrals to a specialist ME/CFS clinic revealed a 37% rejection rate owing to inappropriate referral and of those, 61% had a likely alternative diagnosis.^42^

### Australian women participating in an online survey experience on average a 4 years wait for rediagnosis of their chronic condition

The median time to rediagnosis was 4 years. These findings are in keeping with the current literature. A large 2019 population study at the University of Copenhagen demonstrated that men are diagnosed with chronic conditions at comparatively younger ages than women, and that women on average waited 2–5 years longer than men to obtain a diagnosis.^32^ Rare or chronic conditions take on average 7.6 years to diagnose across both women and men in the United States of America and 5.6 years in the U.K., with patients visiting an average of eight physicians and receiving two to three misdiagnoses.^43^ There are however some areas that appear to have improved in this regard. A 2015 Danish study of autoimmune arthritis demonstrated a significant but decreasing time from presentation to diagnosis from 29 to 66 months in the year 2000 to 3 to 4 months by 2011.^44^ The discrepancy in diagnostic waiting times extends across all medical specialties even to Oncology, where even with higher cancer screening rates,^45^ women have longer diagnostic intervals for several cancers, including, but not limited to: bladder, colorectal, gastric, head and neck, lung, and lymphoma.^46–48^

Our findings of a lengthy time to a final diagnosis in women are possibly a sign of a sex and gender gap in medical care. Studies exploring specific chronic conditions have demonstrated that there is potentially a sex and gender difference in the time from presentation to a doctor with symptoms to receiving diagnosis. Hudson et al assessed the wait time in Canada between onset of Reynaud's phenomenon and the diagnosis of systemic sclerosis and diffuse cutaneous systemic sclerosis in female and male patients, determining that women's wait was significantly longer than that of men, however following other manifestations of illness, this wait time decreased to insignificance.^49^ This suggests support for the Yentl syndrome; a woman must prove herself at least as sick, if not more sick, than a male counterpart to receive diagnosis and treatment.^50^

The findings of this study suggest that women with female or female-dominated conditions experience a lengthy time to final diagnosis. In keeping with this, a 1996 study from the United States of America and United Kingdom demonstrated a diagnostic delay from presentation with symptoms to diagnosis of endometriosis to be an average of 11.73 and 7.96 years, respectively.^51^ Recent reports state that although this time is decreasing, the average wait time for diagnosis of this painful gynecological condition is still between 4 and 11 years.^52,53^ Similarly, despite lupus being a condition that predominantly affects females, a U.K. survey study reported a longer time to diagnosis in women than men (6.9 vs. 4.5 years, respectively).^54^ Women's protracted diagnostic journey for lupus also frequently included doctors diagnosing their symptoms as medically unexplained or psychosomatic.^54^

It is unclear why there is a lengthy duration among presentation, diagnosis, and subsequent rediagnosis in this study. Other studies have suggested that diagnostic delays may be, in part, due to patient anxiety and avoidance of medical appointments. A 2010 survey demonstrated that 38% of patients were afraid of their doctor not taking their symptoms seriously, thus delaying diagnosis.^55^ Despite observation of diagnostic delays in female-predominant conditions, surprisingly little work has been done to quantify this delay against male diagnostic times. This should be an area of further research, to better understand the sex and gender gaps in medicine and improve women's health.

### Rediagnosis of chronic conditions in Australian women participating in an online survey most commonly follows the patterns of organic–organic and psychogenic–organic

This study found that a third of women who were rediagnosed were originally given a psychological or MUS diagnosis that was later rediagnosed as an organic illness. These findings perhaps signal a propensity to first diagnose women with psychological conditions before seeking the organic cause and have been observed in other studies. Utilizing a sample of 23 women recruited from online patient forums, Mendelson determined most women had experienced dismissal of their symptoms and refusal to refer them for further investigation, leading to misdiagnoses of lupus as medically unexplained, psychological, or fibromyalgia.^56^ Assessment of 50 patients with an MS diagnoses noted a misdiagnosis rate of 58%, with women particularly likely to have their symptoms initially misattributed to psychiatric conditions or medically unexplained symptoms and men more likely to be offered referral for orthopedic assessment.^57^

A retrospective survey of 107 patients with paroxysmal supraventricular tachycardia demonstrated that symptoms are unrecognized after initial medical examination in 55% of patients and that women were more likely than men to have their symptoms attributed to psychological causes such as panic and anxiety disorders (65% vs. 32% respectively).^58^ POTS is another chronic condition more prevalent in women than men, often associated with other disorders with orthostatic intolerance such as dysautonomia or EDS. POTS is especially common in younger women and it is frequently undiagnosed or misdiagnosed as anxiety for several years before correct diagnosis.^59^

### Limitations

Surveys have several limitations, including participant interpretation of the questions, inflexibility in answering questions, and recall bias. Furthermore, this survey focused on obtaining median times to diagnosis, rates of rediagnosis, and time to rediagnosis, meaning there is potentially a lack of depth surrounding the process in-between initial and final diagnosis. Also, surveys potentially contain skewed data; the cohort of women who volunteered for this study may be those who have had the most positive or the most adverse experiences within the medical system. Consequently, the participants may not be representative of the population of women with chronic conditions in Australia. Additionally, although the sample size exceeded statistical power calculations, is still small, and this must be recognized when considering the results presented. Finally, this study did not include male participants for a comparison. While this study contains results useful for women's health, we would recommend further study to ascertain if times to diagnosis and rates of rediagnosis are similar in men.

## Conclusion

Median wait times for a diagnosis for women in Australia, when factoring in high rates of rediagnosis and time to rediagnosis, are 4 years. The literature suggests women wait longer for a diagnosis compared with men, even for female-predominant conditions. Almost half of the women in this survey reported their condition being rediagnosed and 32% of these women were first diagnosed with a MUS condition before being rediagnosed with an organic condition. In Australia, there has been no comparative work with male participants, therefore it is difficult to confirm a sex-based discrepancy and further research is required. It is important that clinicians are aware of high rediagnosis rates in female patients and understand the potential impact of systemic biases on the diagnostic process for women under their care.

## Supplementary Material

Supplemental data

## Abbreviations Used

CI: confidence interval
EDS: Ehlers–Danlos syndrome
IBS: irritable bowel syndrome
ICD-10: International classification of diseases
IQR: interquartile range
MCAS: mast cell activation syndrome
MCTD: mixed connective tissue disease
ME/CFS: myalgic encephalomyelitis/chronic fatigue syndrome
MSK: musculoskeletal
MUS: medically unexplained syndromes
POTS: postural orthostatic tachycardia syndrome
RA: rheumatoid arthritis
SE: standard error
SLE: systemic lupus
T2DM: type 2 diabetes mellitus
