# Supplementary material for: Self-Reported Time to Diagnosis and Proportions of Rediagnosis in Female Patients with Chronic Conditions in Australia: A Cross-sectional Survey
Source: Womens Health Rep (New Rochelle). 2022 Sep 12;3(1):749–58. doi: 10.1089/whr.2022.0040 (PMC9518795; doi:10.1089/whr.2022.0040)
Supplement: Supplemental data [file Suppl_Data.docx]

**Supplementary Table: Cox Proportional Hazards Model: Time to diagnosis by initial diagnosis, age, income, employment status, State/Territory, Indigenous status and disability status**

| **Diagnosis** | **Coefficient** | **Std. Err.** | **z** | **P>z** | **95% Conf. Interval** |
| --- | --- | --- | --- | --- | --- |
| Type 1 diabetes | 1 | 1 | 1 | 1 | 1 |
| Anxiety | -1.340147 | 0.6140401 | -2.18 | **0.029** | -2.543643 - -0.1366505 |
| IBS | -2.710938 | 0.6254887 | -4.33 | **0.000** | -3.936874 - -1.485003 |
| Tonsillitis | -1.237265 | 0.8739107 | -1.42 | 0.157 | -2.950098 - 0.4755688 |
| Autoimmune arthritis | -1.949613 | 0.5591079 | -3.49 | **0.000** | -3.045444 - -0.8537811 |
| ME/CFS | -2.797071 | 0.5261669 | -5.32 | **0.000** | -3.828339 - -1.765803 |
| Migraine | -2.038876 | 0.5885391 | -3.46 | **0.001** | -3.192392 - -0.885361 |
| Endometriosis | -3.126426 | 0.5360605 | -5.83 | **0.000** | -4.177085 - -2.075766 |
| Fibromyalgia | -2.719947 | 0.5200291 | -5.23 | **0.000** | -3.739185 - -1.700708 |
| Depression | -0.4099745 | 0.5815703 | -0.70 | 0.481 | -1.549831 - 0.7298823 |
| Chronic pain | -2.444011 | 0.6291124 | -3.88 | **0.000** | -3.677049 - -1.210973 |
| Lupus/MCTD | -2.273381 | 0.5347509 | -4.25 | **0.000** | -3.321474 - -1.225289 |
| POTS | -2.618538 | 0.6403519 | -4.09 | **0.000** | -3.873604 - -1.363471 |
| Mental illness | -2.401615 | 0.7263045 | -3.31 | **0.001** | -3.825146 - -0.9780843 |
| Cancers | -3.19151 | 0.287232 | -2.48 | **0.013** | -5.714428 - -0.668572 |
| Neurological | -2.370281 | 0.5639784 | -4.20 | **0.000** | -3.475658 - -1.264903 |
| Gastrological | -2.353943 | 0.6127873 | -3.84 | **0.000** | -3.554984 - -1.152901 |
| Endocrine | -1.122071 | 0.6321984 | -1.77 | 0.076 | -2.361157 - 0.1170155 |
| MSK | -2.311001 | 0.5525364 | -4.18 | **0.000** | -3.393952 - -1.22805 |
| Thyroid | -2.035923 | 0.630024 | -3.23 | **0.001** | -3.270747 - -0.8010983 |
| Gynaecological | -2.448559 | 0.5873454 | -4.17 | **0.000** | -3.599735 - -1.297383 |
| Renal | -1.100082 | 0.9301448 | -1.18 | 0.237 | -2.923132 - 0.7229683 |
| Haematological | 0.1378137 | 0.7231473 | 0.19 | 0.849 | -1.279529 - 1.555156 |
| Miscellaneous | -2.020981 | 0.5668083 | -3.57 | **0.000** | -3.131905- -0.9100569 |
| Infective/viral | -1.66402 | 0.6631686 | -2.51 | **0.012** | -2.963807 - -0.3642332 |
| Respiratory | -0.9536113 | 0.6503845 | -1.47 | 0.143 | -2.228342 - 0.321119 |
| Cardiology | -2.041281 | 0.6679847 | -3.06 | **0.002** | -3.350507 - -0.7320549 |
| **Age (years) at diagnosis** |  |  |  |  |  |
| 18-25 | 1 | 1 | 1 | 1 | 1 |
| 26-35 | -0.1142207 | 0.1788798 | -0.64 | 0.523 | -0.4648187 - 0.2363772 |
| 36-45 | -0.4228682 | 0.219011 | -1.93 | **0.054** | -0.852122 - 0.0063855 |
| 46-55 | 0.3728048 | 0.2982437 | 1.25 | 0.211 | -0.2117421 - 0.9573518 |
| 56-65 | -0.3196987 | 0.4991788 | -0.64 | 0.522 | -1.298071 - 0.6586738 |
| **Weekly household income (AUD)** |  |  |  |  |  |
| $0-743 | 1 | 1 | 1 | 1 | 1 |
| $744-1431 | 0.0109972 | 0.2036187 | 0.05 | 0.957 | -0.388088 - 0.4100825 |
| $1432-2433 | 0.1497865 | 0.214515 | 0.70 | 0.485 | -0.2706551 - 0.5702281 |
| $2434+ | 0.0250699 | 0.2293314 | 0.11 | 0.913 | -0.4244113 - 0.4745511 |
| **Employment** |  |  |  |  |  |
| Employed | 1 | 1 | 1 | 1 | 1 |
| Disability | 0.4835686 | 0.2441818 | 1.98 | **0.048** | 0.0049809 - 0.9621562 |
| Temporary | 0.4095042 | 0.525388 | 0.78 | 0.436 | -0.6202373 - 1.439246 |
| Self | -0.353397 | 0.2675661 | -1.32 | 0.187 | -0.8778169 - 0.171023 |
| Other | 0.3041039 | 0.1817845 | 1.67 | 0.094 | -0.0521873 - 0.660395 |
| **State/Territory of residence** |  |  |  |  |  |
| New South Wales | 1 | 1 | 1 | 1 | 1 |
| Victoria | 0.0102873 | 0.2005402 | 0.05 | 0.959 | -0.3827643 - 0.4033388 |
| Queensland | -0.1195511 | 0.2196371 | -0.54 | 0.586 | -0.5500319 - 0.3109297 |
| Northern Territory | -0.1059509 | 0.5227762 | -0.20 | 0.839 | -1.130573 - 0.9186716 |
| South Australia | 0.057449 | 0.2895484 | 0.20 | 0.843 | -0.5100555 - 0.6249534 |
| West Australia | -0.4017 | 0.3604845 | -1.11 | 0.265 | -1.108327 - 0.3047467 |
| Australian Capital Territory | 0.2183389 | 0.3891928 | 0.56 | 0.575 | -0.5444649 - 0.9811428 |
| Tasmania | -0.2702374 | 0.3914314 | -0.69 | 0.490 | -1.037429 - 0.4969541 |
| **Indigenous Status** |  |  |  |  |  |
| Non-Indigenous | 1 | 1 | 1 | 1 | 1 |
| Aboriginal | -0.1313299 | 0.4380412 | -0.30 | 0.764 | -0.9898749 - 0.7272151 |
| Aboriginal & Torres Strait Islander | -0.8139712 | 1.206539 | -0.67 | 0.500 | -3.178745 - 1.550803 |
| Torres Strait Islander | -1.113063 | 0.8051271 | -1.38 | 0.167 | -2.691083 - 0.4649574 |
| **Disability status** |  |  |  |  |  |
| Disabled | 1 | 1 | 1 | 1 | 1 |
| Unsure | 0.2723727 | 0.1981068 | 1.37 | 0.169 | -0.1159096 - 0.660655 |
| Not disabled | 0.1559574 | 0.2168983 | 0.72 | 0.472 | -0.2691555 - 0.5810703 |

**Supplementary Table: Logistic regression: Re-diagnosis rates by initial diagnosis, age, income, employment, State/Territory of residence, Indigenous status and disability status**

| **Diagnosis** | **Coefficient** | **Std. Err.** | **z** | **P>z** | **95% Conf. Interval** |
| --- | --- | --- | --- | --- | --- |
| Type 1 diabetes | 1 | 1 | 1 | 1 | 1 |
| Anxiety | -0.1340442 | 1.252375 | -0.11 | 0.915 | -2.588655 - 2.320566 |
| IBS | 0.3510873 | 1.257043 | 0.28 | 0.780 | -2.112672 - 2.814847 |
| Autoimmune arthritis | -0.0765181 | 1.197868 | -0.06 | 0.949 | -2.424297 - 2.27126 |
| ME/CFS | 0.0476521 | 1.118327 | 0.04 | 0.966 | -2.144228 - 2.239532 |
| Migraine | 1.637125 | 1.318946 | 1.24 | 0.215 | -0.9479622 - 4.222213 |
| Endometriosis | 0.2491382 | 1.110753 | 0.22 | 0.823 | -1.927897 - 2.426174 |
| Fibromyalgia | -0.5431109 | 1.121382 | -0.48 | 0.628 | -2.740979 - 1.654758 |
| Depression | 0.231708 | 1.204774 | 0.19 | 0.847 | -2.129605 - 2.593021 |
| Chronic pain | 1.917214 | 1.355919 | 1.41 | 0.157 | -0.7403392 - 4.574767 |
| Lupus/MCTD | -0.2443922 | 1.14936 | -0.21 | 0.832 | -2.497096 - 2.008312 |
| POTS | 2.469421 | 1.507637 | 1.64 | 0.101 | -0.485493 - 5.424334 |
| Mental illness | 0.6168758 | 1.302256 | 0.47 | 0.636 | -1.935499 - 3.169251 |
| Cancers | 1.535003 | 2.03915 | 0.75 | 0.452 | -2.461657 - 5.531663 |
| Neurological | -0.3345257 | 1.178022 | -0.28 | 0.776 | -2.643406 - 1.974354 |
| Gastrological | 0.5796472 | 1.23762 | 0.47 | 0.640 | -1.846044 - 3.005338 |
| Endocrine | -0.0194029 | 1.290155 | -0.02 | 0.988 | -2.54806 - 2.509254 |
| MSK | 1.128414 | 1.160604 | 0.97 | 0.331 | -1.146328 - 3.403155 |
| Thyroid | -0.0112384 | 1.205483 | -0.01 | 0.993 | -2.373941 - 2.351464 |
| Dermatological | 0.1554792 | 1.407561 | 0.11 | 0.912 | -2.603289 - 2.914247 |
| Gynaecological | 0.5234007 | 1.221781 | 0.43 | 0.668 | -1.871245 - 2.918047 |
| Renal | 0.605251 | 1.834362 | 0.33 | 0.741 | -2.990033 - 4.200535 |
| Haematological | 0.1754191 | 1.507105 | 0.12 | 0.907 | -2.778452 - 3.129291 |
| Miscellaneous | -0.1559142 | 1.179532 | -0.13 | 0.895 | -2.467755 - 2.155927 |
| Infective/viral | 1.368149 | 1.428901 | 0.96 | 0.338 | -1.432446 - 4.168743 |
| Respiratory | 0.3682056 | 1.34277 | 0.27 | 0.784 | -2.263575 - 2.999987 |
| **Age (years) at diagnosis** |  |  |  |  |  |
| 18-25 | 1 | 1 | 1 | 1 | 1 |
| 26-35 | -0.1099316 | 0.3313735 | -0.33 | 0.740 | -0.759412 - 0.5395485 |
| 36-45 | 0.2871332 | 0.3884154 | 0.74 | 0.460 | -0.474147 - 1.048413 |
| 46-55 | -0.5714871 | 0.5343717 | -1.07 | 0.285 | -1.618836 - 0.4758623 |
| 56-65 | -0.3838744 | 0.9080378 | -0.42 | 0.672 | -2.163596 - 1.395847 |
| **Weekly household income (AUD)** |  |  |  |  |  |
| $0-743 | 1 | 1 | 1 | 1 | 1 |
| $744-1431 | -0.4941456 | 0.3797174 | -1.30 | 0.193 | -1.238378 - 0.2500869 |
| $1432-2433 | -0.3688002 | 0.3979254 | -0.93 | 0.354 | -1.14872 - 0.4111192 |
| $2434+ | -0.5947789 | 0.4212824 | -1.41 | 0.158 | -1.420477 - 0.2309193 |
| **Employment** |  |  |  |  |  |
| Employed | 1 | 1 | 1 | 1 | 1 |
| Disability | -0.2086164 | 0.4600489 | -0.45 | 0.650 | -1.110296 - 0.693063 |
| Temporary | -1.806551 | 1.335181 | -1.35 | 0.176 | -4.423458 - 0.8103562 |
| Self | 0.0094125 | 0.5175128 | 0.02 | 0.985 | -1.004894 - 1.023719 |
| Other | 0.2431778 | 0.3471336 | 0.70 | 0.484 | -0.4371917 - 0.923547 |
| **State/Territory of residence** |  |  |  |  |  |
| New South Wales | 1 | 1 | 1 | 1 | 1 |
| Victoria | -0.1367792 | 0.3580846 | -0.38 | 0.702 | -0.8386121 - 0.565054 |
| Queensland | -0.2896088 | 0.4032254 | -0.72 | 0.473 | -1.079916 - 0.5006984 |
| Northern Territory | -0.4500622 | 1.004319 | -0.45 | 0.654 | -2.418491 - 1.518367 |
| South Australia | -0.4510594 | 0.5500861 | -0.82 | 0.412 | -1.529208 - 0.6270896 |
| West Australia | -0.6256979 | 0.6275863 | -1.00 | 0.319 | -1.855744 - 0.6043487 |
| Australian Capital Territory | -0.3189275 | 0.7074629 | -0.45 | 0.652 | -1.705529 - 1.067674 |
| Tasmania | - 1.67147 | 0.8875957 | -1.88 | 0.060 | -3.411126 - 0.0681854 |
| **Indigenous Status** |  |  |  |  |  |
| Non-Indigenous | 1 | 1 | 1 | 1 | 1 |
| Aboriginal | 0.04373 | 0.785759 | 0.06 | 0.956 | -1.496329 - 1.583789 |
| Torres Strait Islander | 0.6708925 | 1.583725 | 0.42 | 0.672 | -2.433151 - 3.774936 |
| **Disability status** |  |  |  |  |  |
| Disabled | 1 | 1 | 1 | 1 | 1 |
| Unsure | -0.587072 | 0.3599083 | -1.63 | 0.103 | -1.292479 - 0.1183354 |
| Not disabled | -0.6455135 | 0.3994115 | -1.62 | 0.106 | -1.428346 - 0.1373186 |

**Supplementary Table: Cox Proportional Hazards Model: Time to re-diagnosis by initial diagnosis, age, income, employment status, State/Territory, Indigenous status and disability status**

| **Diagnosis** | **Coefficient** | **Std. Err.** | **z** | **P>z** | **95% Conf. Interval** |
| --- | --- | --- | --- | --- | --- |
| Type 1 diabetes | 1 | 1 | 1 | 1 | 1 |
| Anxiety | -1.548715 | 1.709498 | -0.91 | 0.365 | -4.899269 - 1.801839 |
| IBS | -2.10012 | 1.818582 | -1.15 | 0.248 | -5.664475 - 1.464236 |
| Autoimmune arthritis | -1.302561 | 1.721978 | -0.76 | 0.449 | -4.677576 - 2.072455 |
| ME/CFS | -1.496968 | 1.766967 | -0.85 | 0.397 | -4.96016 - 1.966223 |
| Migraine | -0.2792046 | 1.780403 | -0.16 | 0.875 | -3.768729 - 3.21032 |
| Endometriosis | -1.250097 | 1.726791 | -0.72 | 0.469 | -4.634546 - 2.134352 |
| Fibromyalgia | -1.110412 | 1.759113 | -0.63 | 0.528 | -4.558211 - 2.337386 |
| Depression | -1.658441 | 1.733496 | -0.96 | 0.339 | -5.05603 - 1.739149 |
| Chronic pain | -0.149883 | 1.688589 | -0.09 | 0.929 | -3.459456 - 3.15969 |
| Lupus/MCTD | -1.762891 | 1.723587 | -1.02 | 0.306 | -5.141059 - 1.615278 |
| POTS | -0.8936385 | 1.739835 | -0.51 | 0.608 | -4.303652 - 2.516375 |
| Mental illness | -0.8949151 | 1.395106 | -0.64 | 0.521 | -3.629272 - 1.839442 |
| Cancers | 1.841628 | 3.651833 | 0.50 | 0.614 | -5.315834 - 8.99909 |
| Neurological | -1.127938 | 1.759427 | -0.64 | 0.521 | -4.576352 - 2.320475 |
| Gastrological | 0.2255261 | 1.692092 | -0.13 | 0.894 | -3.090914 - 3.541966 |
| Endocrine | -0.5231696 | 1.907756 | -0.27 | 0.784 | -4.262303 - 3.215964 |
| MSK | -0.7928909 | 1.671181 | -0.47 | 0.635 | -4.068345 - 2.482564 |
| Thyroid | -0.1748463 | 1.811428 | -0.10 | 0.923 | -3.72518 - 3.375487 |
| Dermatological | 0.9803836 | 1.98008 | 0.50 | 0.621 | -2.900502 - 4.861269 |
| Gynaecological | -1.21193 | 1.797077 | -0.67 | 0.500 | -4.734137 - 2.310277 |
| Renal | 2.615947 | 2.330163 | 1.12 | 0.262 | -1.951089 - 7.182983 |
| Haematological | 3.222211 | 1.90542 | 1.69 | 0.091 | -0.5123438 - 6.956766 |
| Miscellaneous | -1.639301 | 1.772931 | -0.92 | 0.355 | -5.114182 - 1.835581 |
| Infective/viral | -1.099417 | 1.811232 | -0.61 | 0.544 | -4.649367 - 2.450532 |
| Respiratory | -0.215362 | 0.078948 | -0.10 | 0.917 | -4.290023 - 3.859303 |
| Cardiology | -2.364074 | 2.150113 | -1.10 | 0.272 | -6.578219 - 1.85007 |
| **Age (years) at diagnosis** |  |  |  |  |  |
| 18-25 | 1 | 1 | 1 | 1 | 1 |
| 26-35 | -0.034429 | 0.3270116 | -0.11 | 0.916 | -0.67536 - 0.6065021 |
| 36-45 | -0.4696796 | 0.3870512 | -1.21 | 0.225 | -1.228286 - 0.2889268 |
| 46-55 | -0.4473623 | 0.5659464 | -0.79 | 0.429 | -1.556597 - 0.6618722 |
| 56-65 | -0.9999764 | 1.248079 | -0.80 | 0.423 | -3.446166 - 1.446213 |
| **Weekly household income (AUD)** |  |  |  |  |  |
| $0-743 | 1 | 1 | 1 | 1 | 1 |
| $744-1431 | -0.1588396 | 0. 4559175 | -0.35 | 0.728 | -1.052421 - 0.7347423 |
| $1432-2433 | -0.2303388 | 0.3880934 | -0.59 | 0.553 | -0.9909879 - 0.530310 |
| $2434+ | -0.4657359 | 0.4415365 | -1.05 | 0.292 | -1.331131 - 0.3996597 |
| **Employment** |  |  |  |  |  |
| Employed | 1 | 1 | 1 | 1 | 1 |
| Disability | -0.2248043 | 0.4218441 | -0.53 | 0.594 | -1.051604 - 0.6019951 |
| Temporary | -3.569777 | 3.080342 | -1.16 | 0.247 | -9.607137 - 2.467584 |
| Self | -0.6035291 | 0.5119879 | -1.18 | 0.238 | -1.607007 - 0.3999488 |
| Other | 0.018774 | 0.4937403 | 0.04 | 0.970 | -0.9489391 - 0.986487 |
| **State/Territory of residence** |  |  |  |  |  |
| New South Wales | 1 | 1 | 1 | 1 | 1 |
| Victoria | -0.0055228 | 0.3604077 | -0.02 | 0.988 | -0.7119089 - 0.700863 |
| Northern Territory | -0.587751 | 1.12702 | -0.52 | 0.602 | -2.79667 - 1.621168 |
| South Australia | -0.5497808 | 0.5480669 | -1.00 | 0.316 | -1.623972 - 0.5244106 |
| West Australia | -0.7129653 | 0.6415619 | -1.11 | 0.266 | -1.970403 - 0.5444729 |
| Australian Capital Territory | -0.4066569 | 0.6731827 | -0.60 | 0.546 | -1.726071 - 0.9127569 |
| Tasmania | -1.219491 | 0.9092168 | -1.34 | 0.180 | -3.001523 - 0.5625415 |
| **Indigenous Status** |  |  |  |  |  |
| Non-Indigenous | 1 | 1 | 1 | 1 | 1 |
| Aboriginal | -0.0793209 | 0.7021657 | -0.11 | 0.910 | -1.45554 - 1.296899 |
| Aboriginal & Torres Strait Islander | -0.405854 | 1.264886 | -0.32 | 0.748 | -2.884985 - 2.073277 |
| Torres Strait Islander | 4.204948 | 1.527405 | 2.75 | **0.006** | 1.21129 - 7.198606 |
| **Disability status** |  |  |  |  |  |
| Disabled | 1 | 1 | 1 | 1 | 1 |
| Not disabled | 0.630847 | 0.345632 | 1.83 | 0.068 | -0.0465792 - 1.308273 |
